# Metal–ligand covalency of C–H activating iridium complexes from L-edge valence-to-core resonant inelastic X-ray scattering

**DOI:** 10.1039/d5sc09924b

**Published:** 2026-02-16

**Authors:** Raphael M. Jay, Ambar Banerjee, Marco Reinhard, Huan Zhao, Nils Huse, Kelly J. Gaffney, Thomas Kroll, Dimosthenis Sokaras, Philippe Wernet

**Affiliations:** a Department of Physics and Astronomy, Uppsala University 75120 Uppsala Sweden raphael.jay@physics.uu.se philippe.wernet@physics.uu.se; b Research Institute for Sustainable Energy (RISE), TCG Centres for Research and Education in Science and Technology (TCG-CREST) Kolkata 700091 India ambar.banerjee@tcgcrest.org; c Stanford Synchrotron Radiation Lightsource, SLAC National Accelerator Laboratory Menlo Park California 94025 USA; d Center for Free-Electron Laser Science, Department of Physics, University of Hamburg 22761 Hamburg Germany; e Stanford PULSE Institute, SLAC National Accelerator Laboratory Menlo Park California 94025 USA

## Abstract

The electronic structure of iridium carbonyl complexes is known to be fundamental to their ability to activate alkane C–H bonds following UV photolysis. Here, we investigate three prototypical iridium complexes with different ancillary ligands using valence-to-core resonant inelastic X-ray scattering measurements at the Ir L_3_-edge in combination with optical absorption spectroscopy and calculations based on time-dependent density functional theory. We characterize experimentally how the nature and degree of metal–ligand hybridization impact valence-excited state energetics as well as how changes in ionic *vs.* covalent metal–ligand interactions for different ancillary ligands modulate charge densities at the central metal atom. The selectivity of our methods to the valence-excited state manifold allows us to observe and quantify shifts in the d–d and charge-transfer manifold of excited-states, which are both thought to influence the yield of photochemical C–H bond activation. Our combined experimental and theoretical study of this series of iridium complexes reveals the interplay of ligand structure, metal–ligand bonding or covalency and valence-excited state landscape, which allows to deduce a general understanding of how these properties impact photochemical pathways and reactivity in C–H activation and other photocatalytic applications.

## Abstract 2

Valence-to-core resonant inelastic X-ray scattering at the metal L_3_-edge reveals how differences in ionic *versus* covalent metal–ligand bonding modulates local metal charge densities and excited-state landscapes in a series of iridium complexes.

## Graphical abstract

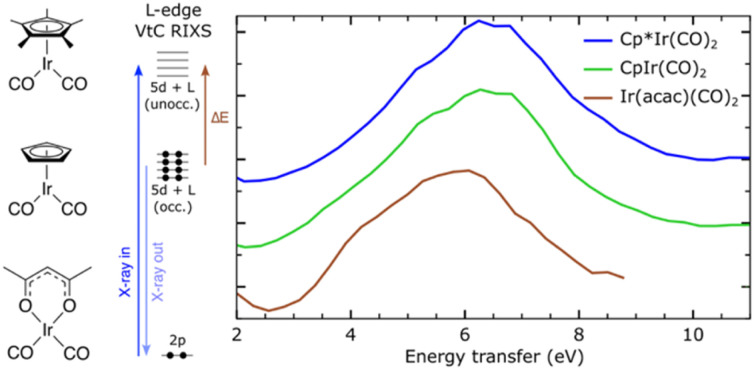

## Introduction

**Fig. 1:**
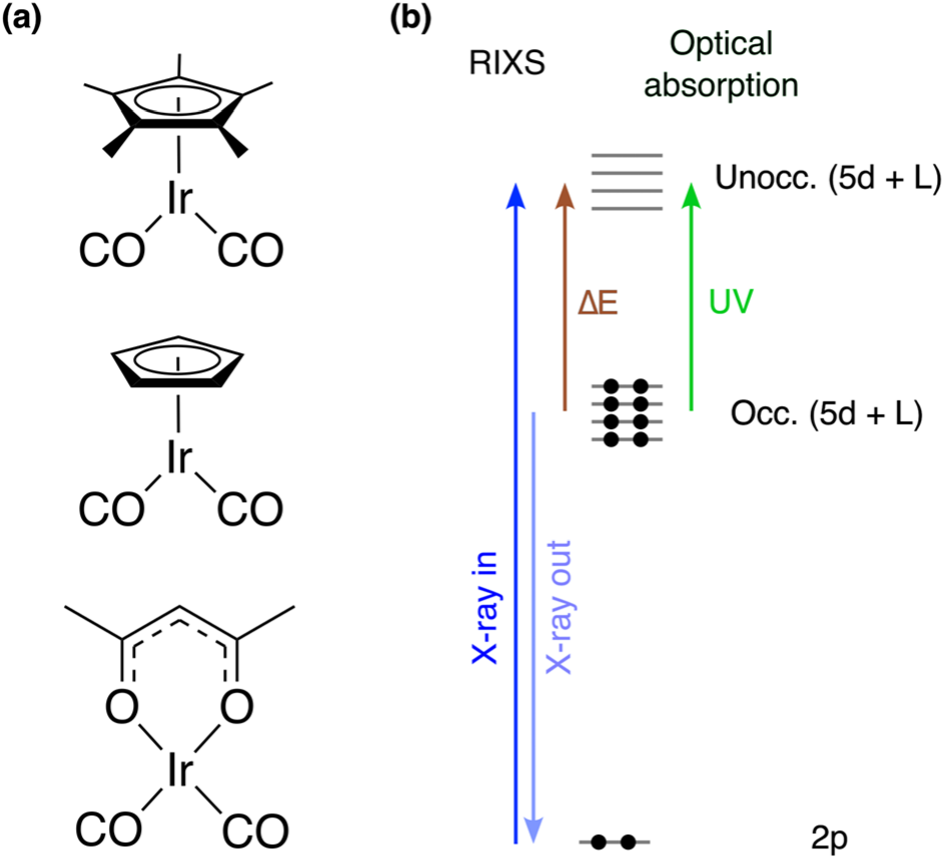
(a) Structures of the complexes studied here: Cp*Ir(CO)_2_ (top), Cp*Ir(CO)_2_ (middle) and Ir(acac)(CO)_2_ (bottom). (b) Schematic of the L-edge VtC RIXS process compared to optical absorption. The metal 2p core-excitation (X-ray in) into unoccupied orbitals of mixed 5d and ligand L character can be approximated by being followed by emission (X-ray out) from occupied orbitals resulting in valence-excited final states of d–d and charge-transfer character (as probed by energy transfer Δ*E*).

Selectively and efficiently cleaving C–H bonds of saturated hydrocarbons for further functionalization is considered one of the grand challenges in chemistry.^1–4^ Following the observation forty years ago that alkane C–H bonds can be activated using photochemically prepared transition metal complexes,^5,6^ photochemical C–H bond activation emerged as a new field. Irradiation of transition metal complexes of the form LM(CO)_2_ (L = Cp/Cp*, Cp = cyclopentadienyl, Cp* = pentamethylcyclopentadienyl, M = Ir, Rh, see Fig. 1a) with ultraviolet (UV) light in an alkane solution was found to induce the loss of a carbonyl (CO) group to form a highly reactive LM(CO) metal-monocarbonyl that ultimately leads to the formation of alkyl hydride species, in which the alkane C–H bond had been broken *via* oxidative addition.^7^

A series of time-resolved studies affirmed the hypothesized reaction pathways and unambiguously identified the decisive intermediates, so-called σ-complexes,^8,9^ on pico- to nanosecond timescales.^10–20^ Using femtosecond optical,^21–24^ infrared^12,20,25–27^ and, more recently, X-ray spectroscopy,^28–30^ CO dissociation was found to occur within several 100 femtoseconds and to be the decisive initial step for C–H activation. It was also observed that the specific structure of the ancillary ligands influences the yield of C–H activation.^18,27,31^ For LM(CO)_2_ complexes, specifically, time-resolved optical spectroscopy studies observed reduced C–H activation for Cp*Ir(CO)_2_ and Cp*Rh(CO)_2_ compared to CpIr(CO)_2_ and CpRh(CO)_2_, which was attributed to a lower yield of the initial CO photodissociation.^31^ No photodissociation at all was even observed when replacing the Cp*/Cp moieties with an acac group^18^ (acac = acetylacetonate, see Fig. 1a). Understanding what determines CO dissociation and what potentially limits the formation of reactive metal-monocarbonyl species is thus essential to understand how to control the first step in photochemical C–H activation with metal-carbonyl complexes.

CO dissociation from metal complexes is generally thought to proceed *via* an initial metal-to-ligand charge-transfer (MLCT) excitation of the system followed by a rapid population of dissociative d–d states.^32–34^ The relative arrangement of d–d *vs.* MLCT valence-excited states strongly depends on the specific ligand structure as well as the resulting ligand field strength and degree of metal–ligand covalency. Understanding the exact energetic ordering of d–d *vs.* MLCT states therefore helps in understanding what influences the efficiency of photochemical C–H activation. Direct experimental access to the full valence-excited state landscape including d–d states, however, poses significant challenges. Determining the energy of d–d excited states can be challenging with conventional methods, because d–d states are generally optically dark due to spin and parity selection rules and thus only constitute minor contributions to the optical absorption spectrum.^35–37^ Additionally, d–d transitions are often buried under much more intense charge-transfer excitations in the UV-visible absorption spectrum. When strong-field ligands are involved, d–d states are also often located deep in the UV range where absorption measurements prove difficult.

Here, we employ a combination of optical absorption spectroscopy, Ir L_3_-edge X-ray absorption spectroscopy (XAS) and valence-to-core (VtC) resonant inelastic X-ray scattering (RIXS) at the Ir L_3_-edge to map the d–d and MLCT states of Cp*Ir(CO)_2_, CpIr(CO)_2_ and Ir(acac)(CO)_2_. As the X-ray analogue of resonant Raman spectroscopy, RIXS obeys different dipole selection rules than optical spectroscopy^36^ and is ideally suited to complement optical spectroscopy. A scheme of the L-edge VtC RIXS process is displayed in Fig. 1b. The initial core-excitation elevates a metal 2p electron into unoccupied metal d-derived orbitals as well as ligand orbitals, which hybridize with metal d orbitals and thereby adopt metal d character (2p → 5d dipole transitions). The resulting core-hole can be filled by core or valence electrons from lower-lying occupied orbitals in a resonant emission process. By collecting the VtC emission resulting from the transitions of valence electrons from the occupied 5d-derived molecular orbitals (5d → 2p dipole transitions), the final states probed by this 2p–5d VtC RIXS process are valence-excited states with predominantly d–d and charge-transfer character. This approach is therefore analogous to 2p–3d RIXS experiments, which have been widely used to characterize the d–d manifold of 3d transition metal complexes.^28,29,37–44^ Recently, similar studies based on 2p–4d VtC RIXS have also become feasible for 4d metal complexes.^45–47^ Studies on 5d metal complexes based on 2p–5d VtC RIXS have remained scarce to date.^48,49^

Here, we use the sensitivity of the method to experimentally evaluate how the differences in ligand structure of Cp*Ir(CO)_2_, CpIr(CO)_2_ and Ir(acac)(CO)_2_ modulate local charge densities at the metal and influence the respective d–d *vs.* MLCT manifolds. Our RIXS measurements are complemented by quantum chemical simulations of the optical absorption and Ir L_3_-edge VtC-RIXS spectra based on time-dependent density functional theory (TD-DFT). This combination of experimental and theoretical insights provides a complete picture of how the different ligands of the studied Ir carbonyl complexes change their valence-excited state manifolds as well as local charge densities at the metal center and how this can affect photochemical pathways and reactivity in C–H bond activation and other photocatalytic applications with transition metal carbonyls.

## Methods

### Materials

Cp*Ir(CO)_2_ and CpIr(CO)_2_ were purchased from HetCat, Ir(acac)(CO)_2_ from Sigma-Aldrich. Cp*Ir(CO)_2_ and CpIr(CO)_2_ were prepared as ∼10 mM, Ir(acac)(CO)_2_ as ∼2 mM solutions in cyclohexane.

### Experimental details

The X-ray spectroscopy measurements were conducted at the SSRL synchrotron radiation facility using the beamline 15-2. The incident photon energy was tuned across the Ir L_3_-edge using a Si(311) crystal monochromator. The incident photon flux was ∼4 × 10^12^ photons per s. The incident photon energy was energy-calibrated with respect to the Pt L_3_-edge using a Pt foil. The bandwidth of the incident photon energy was ∼0.3 eV. The X-ray spot size on the sample was 38 µm (horizontal) × 6 µm (vertical). The VtC X-ray emission was collected across the Ir L_3_-edge using a Johann-spectrometer in Rowland-geometry, in which a set of five spherically bent Si(844) crystals collect the emitted X-ray photons and focus them on the detector. The energy axis of the spectrometer was calibrated using the elastically scattered incidence X-rays. The spectrometer resolution was 1.1 eV as estimated by fitting the elastic line at incident photon energies below the onset of the Ir L_3_-edge. X-ray absorption (XAS) measurements were performed in high energy-resolution fluorescence detection mode (HERFD) by collecting the Ir Lα_1_ fluorescence across the Ir L_3_-edge using a single Si(800) spherically bent crystal. The HERFD spectra were extracted as a cut through the fluorescence map at a constant emission energy of ∼9175 eV. The liquid samples were delivered into the interaction zone *via* a 1 mm cylindrical jet driven by an HPLC pump at a flow rate of 40 ml min^−1^. The sample was collected below the interaction zone and pumped back into the sample reservoir *via* a peristaltic pump. HERFD-XAS measurements at the Ir L_3_-edge were conducted periodically to monitor potential sample degradation.

### Computational details

All calculations have been performed using the ORCA 5.0.2 quantum chemistry package.^50^ The molecular structures of Cp*Ir(CO)_2_, CpIr(CO)_2_ and Ir(acac)(CO)_2_ (see SI) were optimized using the TPSSh^51^ functional and the def2-TZVPP basis set.^52^ The energetic minima of the optimized structures were confirmed using vibrational normal mode frequency calculations. All calculations used the RIJCOSX approximation^53^ for computational efficiency and a conductor-like polarizable continuum model^54^ (CPCM) to implicitly account for effects of the cyclohexane solvent. Molecular orbital decompositions in terms of their atomic character were performed using Mulliken analysis, while the coordinate system adopted for identifying individual metal d orbitals was based on the coordinate system of the Ir(CO)_2_ structural motif common to all three complexes (see SI).

Valence-excited states were computed using linear response time-dependent density functional theory (TD-DFT) at the B3LYP^55,56^ level of theory using the ZORA approximation^57^ to account for relativistic effects. For the Ir atom, the SARC-ZORA-TZVPP basis set^58^ was used, whereas the ZORA-def2-TZVPP was used for all other atoms. UV/visible absorption spectra were then generated by convolving the calculated optical transition dipole moments with 0.5 eV full width half max (FWHM) Gaussian functions to account for experimental and conformational broadening.

Ir L_3_-edge VtC RIXS spectra were calculated using the restricted orbital subspace TD-DFT method recently established by Nascimento *et al.*^46^ and Vaz da Cruz *et al.*^59^ The three Ir 2p orbitals are included in the active orbital subspace, next to the 21 highest occupied orbitals as well as the 20 lowest unoccupied orbitals. RIXS intensities were then calculated by solving 60 (3 × 20) core-excited intermediate states as well as 420 (21 × 20) valence-excited final states using the Kramers–Heisenberg formalism implemented in the Multi-Wfn program package.^60^ For CpIr(CO)_2_ and Ir(acac)(CO)_2_, this covered the energy transfer axis up to ∼15 eV as probed in the experiment. Due to the higher numbers of atoms in Cp*Ir(CO)_2_, the same number of excitations only covered the energy transfer range up to ∼10 eV. Due to the associated computational cost, the number of computed excitations could not be increased for Cp*Ir(CO)_2_. In the direction of the incident photon energy, the calculated VtC-RIXS transitions are convolved with Pseudo-Voigt functions including a Lorentzian broadening of 5.24 eV FWHM to account for the lifetime broadening of the Ir 2p core-levels^61^ and a Gaussian broadening of 0.6 eV FWHM to account for experimental and conformational broadening. In the direction of the energy transfer, a Gaussian broadening of 1.1 eV FWHM is applied to account for the spectrometer resolution convolved with an additional 0.6 eV broadening to account for experimental and conformational broadening.

## Results and discussion

**Fig. 2:**
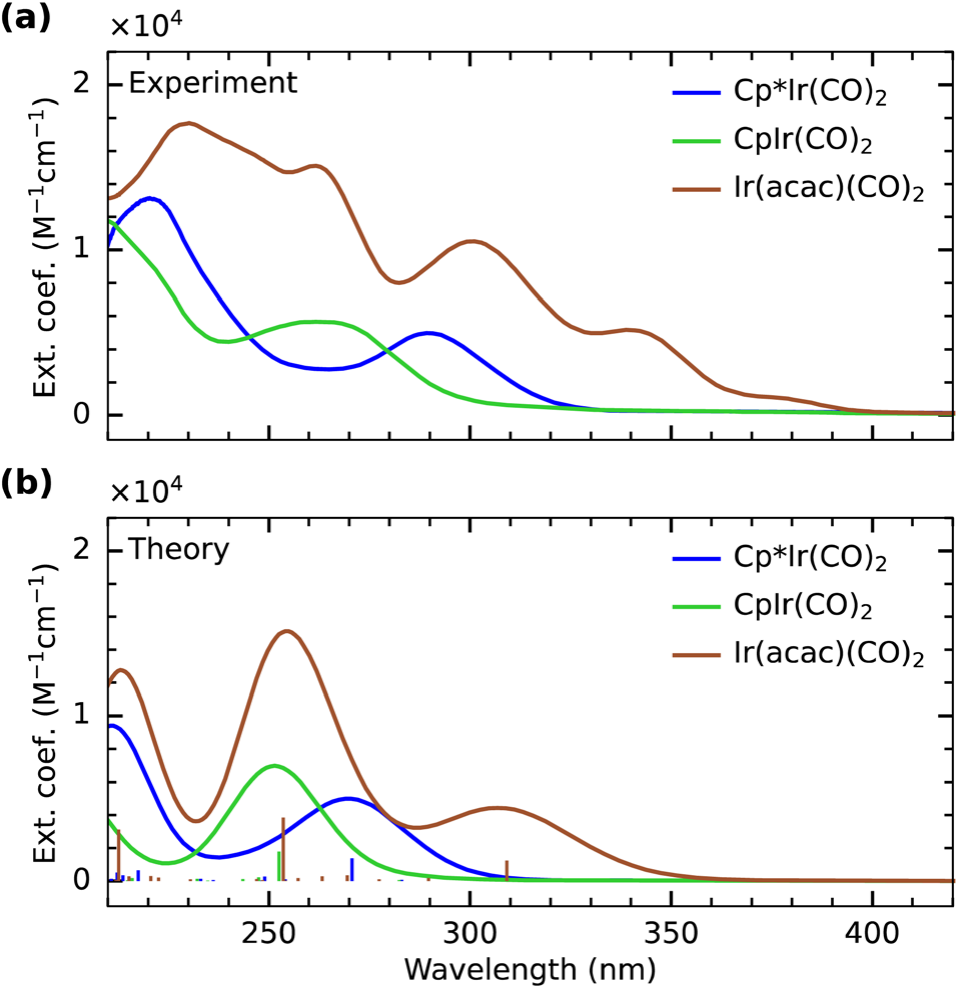
(a) Experimental and (b) calculated UV/visible absorption spectra of Cp*Ir(CO)_2_, CpIr(CO)_2_ and Ir(acac)(CO)_2_. The calculated spectrum of Cp*Ir(CO)_2_ is scaled so that the intensity of the lowest-energy absorption band matches the intensity of the experimental spectrum. All other calculated spectra are scaled accordingly.

In Fig. 2, we show the experimental and calculated UV/Vis absorption spectra of Cp*Ir(CO)_2_, CpIr(CO)_2_ and Ir(acac)(CO)_2_. The intensity of the lowest-energy absorption band in the calculated spectrum of Cp*Ir(CO)_2_ is scaled to match the intensity of the experimental absorption band. The same scaling is applied to the calculated spectra of the other two complexes. In experiment (Fig. 2a), the lowest-energy absorption bands of CpIr(CO)_2_ and Cp*Ir(CO)_2_ are centered at 265 and 290 nm, respectively. Despite some deviations in absolute energies, the experimentally observed relative shift of 0.4 eV between the two complexes is well reproduced by the calculations shown in Fig. 2b, which exhibit a shift of 0.35 eV. The transitions underlying these lowest-energy absorption bands can be clearly assigned to Ir (5d) → CO (π*) MLCT excitations (see plots of the involved Kohn–Sham orbitals in the SI) in agreement with the general notion that UV excitation of metal carbonyls induces MLCT excitations.^33^ The observed shift in excitation energy therefore indicates that the MLCT excited-state energy as measured here in the Franck–Condon region is lower by 0.4 eV in Cp*Ir(CO)_2_ compared to CpIr(CO)_2_. This difference can be rationalized by considering the effect of Cp methylation on the charge distribution within the complex. Methyl groups are known to act as electron-donating moieties leading to an increase in electron donation and thus higher electronic charge density at the iridium center.^5^ A more electron-rich metal center is then expected to exhibit a higher degree of metal-to-ligand charge delocalization through an increase in Ir (5d) → CO (π*) back-donation.^62^ Increased back-donation in turn reduces the energy between the Ir (5d) and CO (π*) orbitals and thus decreases the energy of the associated transitions as observed here.

In contrast to Cp*Ir(CO)_2_ and CpIr(CO)_2_, the spectral features of Ir(acac)(CO)_2_ are only poorly reproduced by the TD-DFT calculations (Fig. 2a and b). While the individual experimental transitions are thus challenging to assign, our calculations indicate characteristic differences in the optical spectrum of Ir(acac)(CO)_2_ with respect to the spectra of Cp*Ir(CO)_2_ and CpIr(CO)_2_ (see plots of the involved Kohn–Sham orbitals in the SI). Our calculations suggest that the lowest energy absorption band at 290–340 nm in Ir(acac)(CO)_2_ constitutes an MLCT excitation involving the promotion of an electron to primarily CO π* character ligand orbitals. In Cp*Ir(CO)_2_ and CpIr(CO)_2_, these lowest-energy absorption bands involve Ir 5d_*yz*_-derived orbitals, whereas in Ir(acac)(CO)_2_, the excitation is from the Ir 5d_*z*^2^_-derived orbital. In Cp*Ir(CO)_2_ and CpIr(CO)_2_, the analogous MLCT excitations involving the Ir 5d_*z*^2^_-derived orbitals are at significantly higher energies in the range of 240–250 nm. This substantial lowering of Ir (5d) → CO (π*) MLCT transitions in Ir(acac)(CO)_2_ can be rationalized by the ionic character of the iridium–acac bond. The strong electron affinity of the acac ligands results in a charge-depleted Ir center and in turn a reduction in back-donation from Ir into the CO (π*) system. This lowers the energy separation of Ir(5d) and CO (π*) orbitals for Ir(acac)(CO)_2_ compared to the more covalent Cp*Ir(CO)_2_ and CpIr(CO)_2_ complexes. The calculations further suggest MLCT excitations into the acac (π*) manifold in Ir(acac)(CO)_2_ to appear in the range of 240–270 nm. For Cp*Ir(CO)_2_ and CpIr(CO)_2_, in contrast, transitions into the Cp/Cp* π* orbitals are found to occur at higher energy in the range of 200–250 nm.

**Fig. 3:**
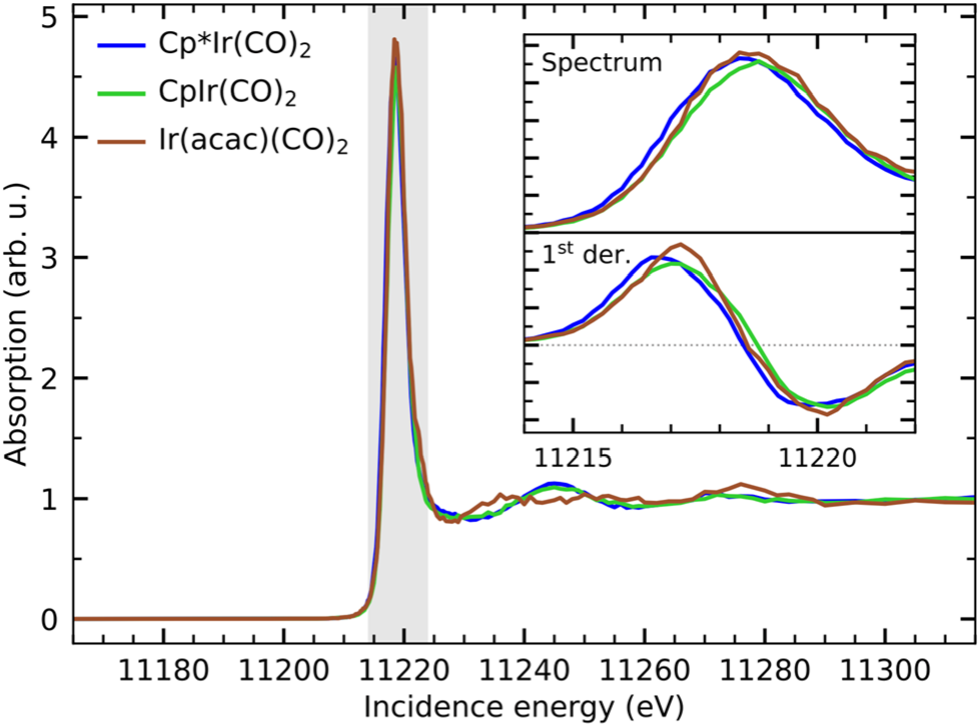
HERFD X-ray absorption spectra of Cp*Ir(CO)_2_, CpIr(CO)_2_ and Ir(acac)(CO)_2_ measured at the Ir L_3_-edge. The spectra are normalized to the edge-jump at 11 500 eV photon energy. The inset shows a closeup of the absorption around the whiteline intensity (shaded area) as well as its 1st derivative.

In Fig. 3, we show the Ir L_3_-edge HERFD X-ray absorption spectra of Cp*Ir(CO)_2_, CpIr(CO)_2_ and Ir(acac)(CO)_2_. The spectra are very similar with a strong whiteline absorption just below 11 220 eV. When zooming in on the whiteline, as shown in the inset in Fig. 3, minor variations of peak height and peak position can be observed. The integrated whiteline intensity of Ir(acac)(CO)_2_ is ∼8% higher than that of Cp*Ir(CO)_2_ and CpIr(CO)_2_ (see SI for detailed analysis). This can again be interpreted as a reflection of the more ionic bonding in Ir(acac)(CO)_2_ than in the more covalently bound Cp*Ir(CO)_2_ and CpIr(CO)_2_ complexes. The higher ionicity of Ir(acac)(CO)_2_ results in more atomic-like 5d orbitals with an overall increase in cross section for the 2p → 5d excitations underlying the Ir L_3_-edge whiteline absorption.

The whitelines further exhibit relative shifts with respect to each other, which can be interpreted as a signature of a partial oxidation state change and thus a measure of the varying local charge density at the metal.^38,63–65^ The magnitude of the relative shifts can be quantified by determining the position of the inflection point of the rising edge of the whiteline from its 1st derivative additionally displayed in the inset in Fig. 3 (see SI for detailed analysis). The whiteline of CpIr(CO)_2_ is shifted to higher energy by 0.2 eV with respect to Cp*Ir(CO)_2_, again indicating a lower electronic charge density locally at the metal at CpIr(CO)_2_ with respect to Cp*Ir(CO)_2_. The spectrum of Ir(acac)(CO)_2_ is shifted by another 0.1 eV with respect to CpIr(CO)_2_, indicating an even further decrease in local charge density. This interpretation of Ir L_3_-edge HERFD XAS spectra as well as the interpretation of the optical absorption data is consistent with the calculated Mulliken charges of the three complexes, which suggest that Ir(acac)(CO)_2_ indeed has the highest local (positive) charge at the Ir center, followed by CpIr(CO)_2_ and with Cp*Ir(CO)_2_ having the most electron rich Ir center (*i.e.*, the lowest positive charge at the Ir center, see Table 1).

**Table 1:** Calculated Mulliken charges and literature values of CO stretch frequencies

Complex	Ir Mulliken charge	CO stretch frequencies^a^ (cm^−1^)	
		Symmetric	Anti-symmetric
Cp*Ir(CO)_2_	0.33	2020	1953
CpIr(CO)_2_	0.38	2043	1976
Ir(acac)(CO)_2_	0.51	2074	2000

a Values taken from ref. 12 and 18.

This interpretation of the differences in optical and X-ray absorption spectra in terms of differences in ionic and covalent character and resulting differences in the degree of back-donation into the CO (π*) system is furthermore consistent with the differences in previously reported CO stretching frequencies of the three complexes^12,18^ (see Table 1). Within the framework of the Tolman electronic parameter,^66^ the strongest electron-donating ancillary ligand Cp* in Cp*Ir(CO)_2_ with the highest Ir (5d) → CO (π*) back-donation results in the lowest CO stretch frequencies. Reduction of electron donation onto Ir with Cp and even more with acac then reduces back-donation to CO as reflected in increased CO stretch frequencies in CpIr(CO)_2_ and even more in Ir(acac)(CO)_2_.

**Fig. 4:**
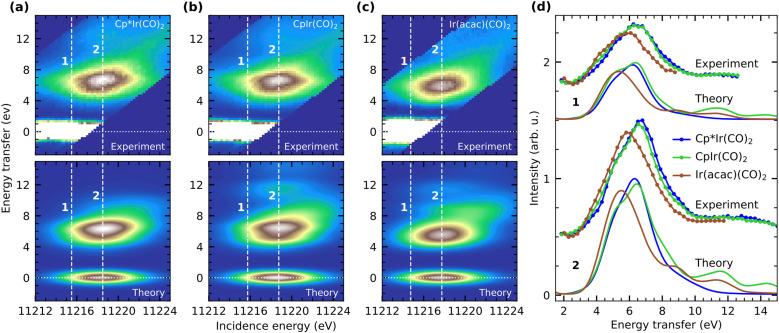
Experimental Ir L_3_ VtC RIXS maps of (a, top) Cp*Ir(CO)_2_, (b, top) CpIr(CO)_2_ and (c, top) Ir(acac)(CO)_2_ measured across the whiteline absorption of the Ir L_3_-edge. The intense elastic scattering (0 eV energy transfer) is saturated to better visualize the weaker inelastic scattering contributions at energy transfers of 3–14 eV. Theoretical Ir L_3_ VtC RIXS maps of (a, bottom) Cp*Ir(CO)_2_, (b, bottom) CpIr(CO)_2_ and (c, bottom) Ir(acac)(CO)_2_ as calculated at the TD-DFT level of theory (vertical white lines mark the incidence energies at which the RIXS spectra shown in panel (d) are extracted). (d) Experimental and calculated RIXS spectra of Cp*Ir(CO)_2_, CpIr(CO)_2_ and Ir(acac)(CO)_2_ as extracted at energies 1 (11 215.50 eV, 11 215.75 eV and 11 214.75 eV, respectively) and 2 (11 218.50 eV, 11 218.75 eV and 11 217.75 eV, respectively).

To understand how these variations in electronic charge distributions are expressed in and can be detailed with the energies of d–d excited-states, we turn to the VtC RIXS data of Cp*Ir(CO)_2_, CpIr(CO)_2_ and Ir(acac)(CO)_2_. In Fig. 4a–c, we show the experimental RIXS maps of the three complexes (top) as recorded by tuning the incident photon energy across the whiteline of the Ir L_3_-edge while simultaneously recording the resonant VtC X-ray emission. Analogously to the X-ray absorption spectra, the RIXS maps of the three complexes are very similar. The spectra exhibit strong elastic scattering (0 eV energy transfer) as well as broad inelastic scattering contributions at energy transfers centered around ∼6.5 eV. Additional inelastic features with lower intensity can be observed at higher energy transfer in the range of ∼12 eV. Due to geometric constraints of the spectrometer, the elastic line moves out of the acceptance range of the spectrometer at higher incident photon energies.

In order to enable not only the comparison of the energies of RIXS transitions but also their intensities, the three individual RIXS maps are normalized to the total acquisition time as well as to the edge-jump at 11 500 eV in the HERFD XAS spectra as a measure of the sample concentration (the HERFD XAS spectra were recorded alternately to single RIXS maps, all HERFD XAS spectra and RIXS maps were then summed). The maximum of the inelastic features of Cp*Ir(CO)_2_ (see Fig. 4a) is scaled to one and the two other RIXS maps are scaled accordingly. The resulting positions, shapes and relative intensities of the inelastic peaks recorded in experiment are well-reproduced by our VtC RIXS calculations (Fig. 4a–c, bottom). To enable this comparison between experiment and theory, the calculated RIXS maps of Cp*Ir(CO)_2_, CpIr(CO)_2_ and Ir(acac)(CO)_2_ are individually shifted along the incidence energy axis by −582 eV, −581.8 eV and −582.5 eV, respectively. This procedure allows to align the maxima of the calculated with those of the experimental RIXS maps and enables a proper comparison between experiment and theory despite the failure of the calculations to reproduce the relative shifts in incidence energy with the needed accuracy. The maximum of the inelastic features of the calculated RIXS map of Cp*Ir(CO)_2_ (see Fig. 4a) is again scaled to one and the other two calculated RIXS maps are scaled accordingly.

For a more detailed comparison between experiment and theory, we turn to the experimental and calculated RIXS spectra of Cp*Ir(CO)_2_, CpIr(CO)_2_ and Ir(acac)(CO)_2_ in Fig. 4d. RIXS spectra were extracted from the RIXS maps at the incidence energies of the respective maxima of the inelastic RIXS features as well as at an incidence energy 3 eV below the maxima (denoted as 2 and 1, respectively, see Fig. 4a–c). The experimental spectra of Cp*Ir(CO)_2_ and CpIr(CO)_2_ extracted at both 1 and 2 are very similar in shape. For the case of Ir(acac)(CO)_2_, however, the peaks of the spectra are shifted by about 1.5 eV to lower energy transfer and are reduced in intensity. These differences in relative energies and intensities are excellently reproduced by our calculated RIXS spectra, which are also extracted at the respective maxima of the inelastic RIXS features as well as at an incidence energy 3 eV below the maxima. This high level of agreement allows for a robust assignment of individual valence-excited final states as well as a detailed analysis of spectral differences between the three complexes.

It is important to note that, due to the short lifetime of the Ir 2p core-hole and the resulting lifetime broadening of 5.2 eV, selected core-excited states within the whiteline of the absorption spectrum cannot be individually accessed by X-ray absorption, even though our incident photon energy bandwidth was 0.3 eV. Instead, the RIXS final states reached in our experiment always result from excitations of multiple core-excited intermediate states. At incident photon energies labeled 2 (Fig. 4a), Ir 2p electrons are dominantly excited to the lone unoccupied 5d-derived orbitals as well as CO (π*) orbitals. Resonant X-ray emission predominantly results from transitions between occupied 5d-derived orbitals to the Ir 2p core hole resulting predominantly in d–d and d → CO π* MLCT transitions in the complexes (Δ*E* in Fig. 1b). The broad (and substantially weaker) features at around 12 eV in our RIXS spectra (Fig. 4d) can instead be assigned to transitions involving occupied lower-lying ligand orbitals. These exhibit some degree of hybridization with the metal d orbitals, but because they generally result in highly mixed ligand-to-metal charge-transfer (LMCT) and ligand–ligand (L–L) RIXS excitations that are difficult to assign and model, we discard them here.

To better isolate contributions from the lower-energy final states, we turn to the RIXS spectra extracted at incident photon energies labeled 1, where the incidence energy is detuned to energies substantially below the whiteline maximum (see Fig. 4). Excitation at 1 reduces the contributions of core excitations into higher-lying ligand orbitals and instead enhances contributions of excitations into orbitals derived from the lone unoccupied 5d orbital. This becomes directly apparent in our RIXS spectra in Fig. 4d, where contributions of the lower-lying transitions at 3–5.5 eV are enhanced with respect to the contributions of the transitions at 5.5–8 eV. At 2, in contrast, the higher-energy transitions dominate.

**Fig. 5:**
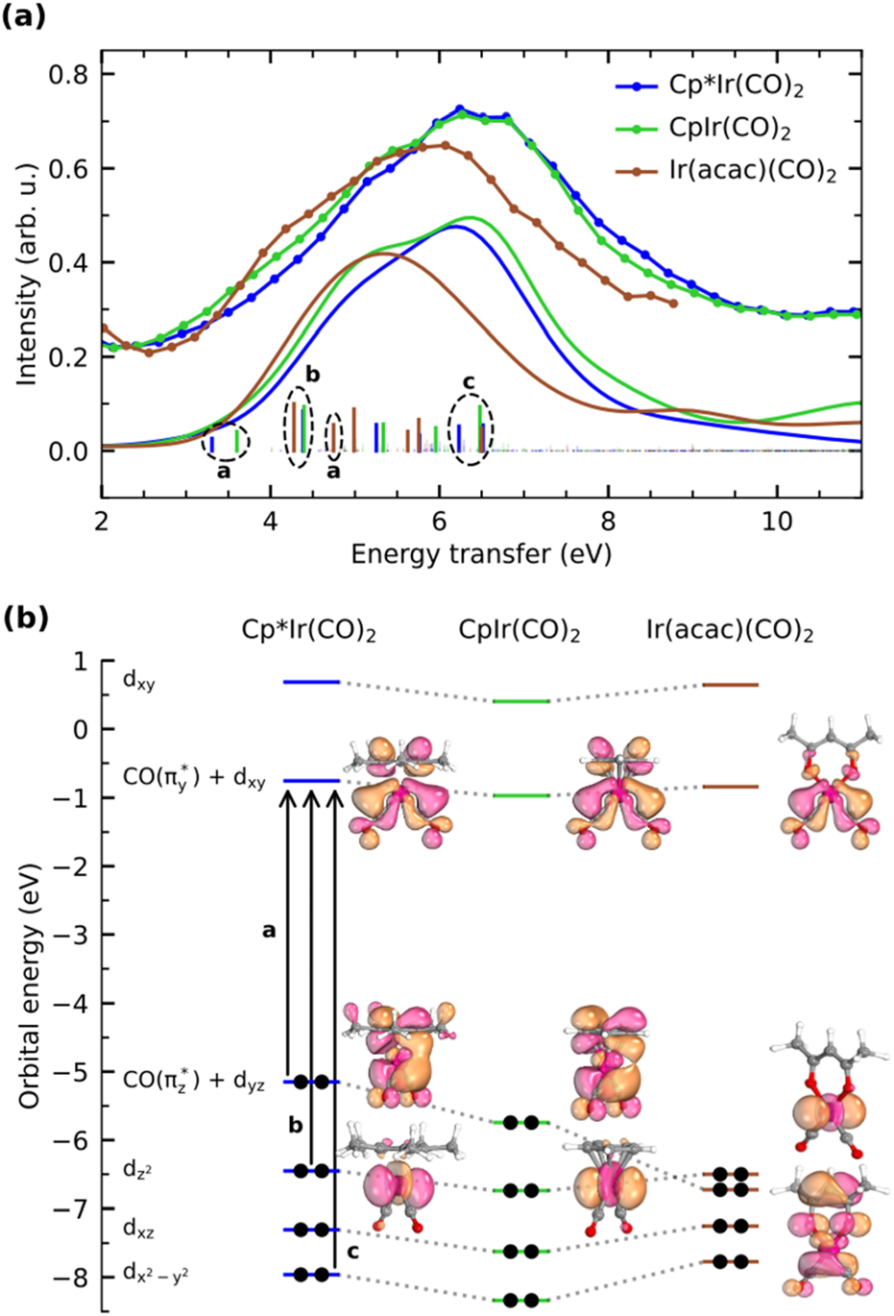
(a) Measured and calculated RIXS spectra of d–d transitions in Cp*Ir(CO)_2_, CpIr(CO)_2_, and Ir(acac)(CO)_2_ (RIXS spectra extracted at incident photon energy 1 as detailed in Fig. 4, individual transitions are shown as sticks, dominant transitions are highlighted with color). (b) Orbital correlation diagram of the Ir 5d orbitals dominantly contributing to the d–d RIXS final states in (a) along with plots of selected Kohn–Sham orbitals (all orbitals are plotted at an isovalue of 0.05, d_*xz*_ and d_*x*^2^−*y*^2^_ are shown in the SI).

With Fig. 5, we now concentrate on the RIXS spectra extracted at 1 and we compare measured and calculated spectra by detailing the calculated individual d–d RIXS transitions underlying the convolved spectra (shown as sticks in Fig. 5a). Strong transitions, which predominantly involve Ir 5d-derived orbitals, are highlighted in color. An orbital correlation diagram of these orbitals is displayed in Fig. 5b. These include the nominal Ir 5d-derived orbitals d_*xy*_, d_*z*^2^_ and d_*x*^2^−*y*^2^_ and d_*xz*_ as well as related orbitals of CO (π*) character with substantial Ir 5d admixture (denoted as 
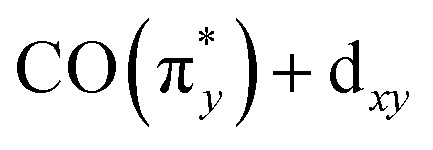
 and 
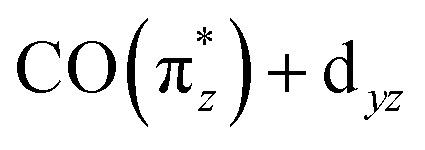
). As shown in Fig. 5b, the calculations allow us to robustly assign the transitions in the range of 3 eV to 4.8 eV to single-electron excitations from the occupied 
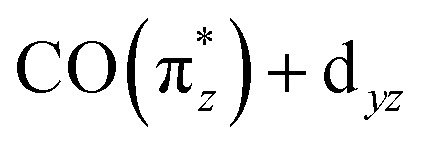
 orbital to the unoccupied 
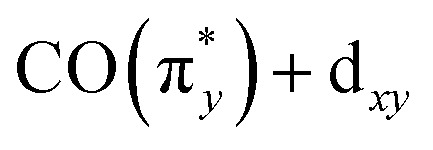
 orbital (denoted as a) as well as from the occupied d_*z*^2^_ orbital to the same unoccupied 
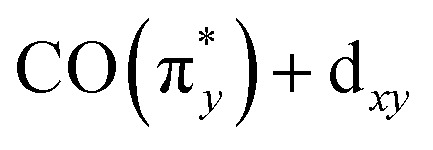
 orbital (denoted as b). Transitions between 4.8 and 6.5 eV are of more mixed character and could only be assigned based on a natural transition orbital (NTO) analysis (see SI for a detailed analysis). In the range of 4.8 to 6 eV, the observed transitions constitute highly mixed excitations from the occupied d_*xz*_ and d_*z*^2^_ orbitals into the unoccupied 
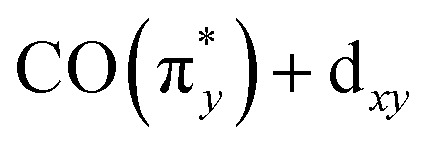
 orbital with additional substantial admixture of unoccupied ligand orbitals, and thus, MLCT character. Based on the NTO analysis, transitions in the range of 6.2 to 6.6 eV (denoted as c) can further be assigned to dominantly correspond to excitations from the occupied d_*x*^2^−*y*^2^_ into the unoccupied 
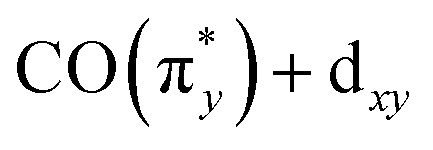
 orbital. Changes of transitions a and b with changes in ligand environment are mainly responsible for the relative shifts of the onset of the RIXS spectra in the range of 3 to 5 eV, whereas differences in transition c are mainly responsible for the substantially lower intensity in the range of 5.5 to 7 eV in Ir(acac)(CO)_2_ with respect to Cp*Ir(CO)_2_ and CpIr(CO)_2_. Transitions beyond an energy transfer of 6.5 eV are of strongly mixed character and low oscillator strength and thus carry limited information on the electronic structure of the complexes.

In more detail, we see in Fig. 5a that the energy of the 
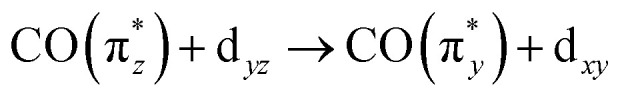
 transition a increases from 3.3 eV in Cp*Ir(CO)_2_ to 3.7 eV in CpIr(CO)_2_, and to 4.7 eV in Ir(acac)(CO)_2_. At the same time and along the Cp*/Cp/acac series, transition a gains substantial amplitude. Because the unoccupied 
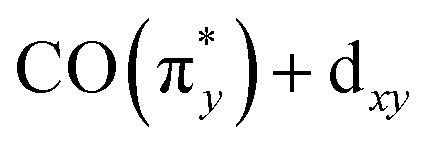
 orbital does not significantly change in character or energy along the Cp*/Cp/acac series (see Fig. 5b), changes of transition a (and, in fact, changes of transition b as well) can fully be rationalized by the changes in energies and charge-transfer character of the highest occupied orbitals of the three complexes. For this, we turn to the results of a fragment charge decomposition analysis given in Table 2. This theoretical analysis provides a measure of the differences in total electronic charge transfer through hybridization between the three ancillary ligands Cp*^−^, Cp^−^ and acac^−^ and the Ir^+^(CO)_2_ moiety. This analysis also allows for detailing the degree of charge transfer that is facilitated *via* the formation of specific molecular orbitals between the fragments. Additionally, an analogous analysis is given for the total charge transfer between the LIr and the (CO)_2_ fragments (where L = Cp*, Cp and acac). The fragment charge decomposition (see Table 2) shows that the amount of total charge transfer from Cp*^−^/Cp^−^ onto Ir^+^(CO)_2_ in Cp*Ir(CO)_2_ and CpIr(CO)_2_ is close to 1. The coordination of the Cp*^−^/Cp^−^ anions to the Ir^+^(CO)_2_ cation thus leads to a level of charge transfer that roughly neutralizes each charge component. Consequently, the character of the bonding between the Cp*/Cp ligands and the Ir center can be inferred to be highly covalent. The corresponding charge transfer between the acac^−^ and the Ir^+^(CO)_2_ moiety in Ir(acac)(CO)_2_ is reduced to 0.7, indicative of its more ionic character. Table 2 further shows the charge transfer from the L^−^ onto the Ir^+^(CO)_2_ specifically along the 
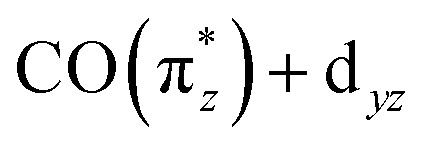
 orbital. In the Cp*/Cp/acac series, the highest charge transfer is observed for the Cp* ligand, indicative of the most substantial hybridization of the 
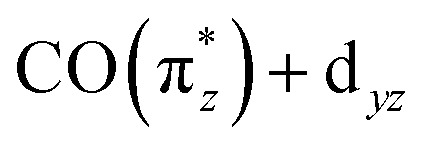
 orbital with the Cp* ligand (this stronger hybridization is also apparent in the plot of the 
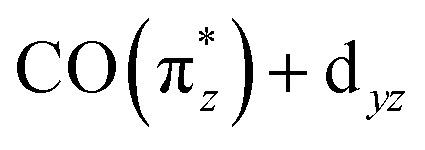
 orbital in Cp*Ir(CO)_2_ in Fig. 5b). The strong hybridization for Cp* corresponds to a low Ir 5d character of this orbital, which is reflected in a comparably low oscillator strength of the associated transition a in Cp*Ir(CO)_2_. Hybridization and associated charge transfer is reduced for the Cp ligand in CpIr(CO)_2_ (see Table 2) leading to a stabilization of the 
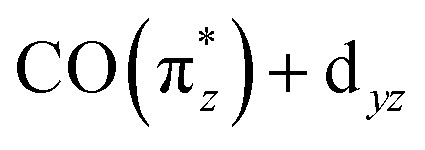
 orbital energy by 0.5 eV compared to Cp*Ir(CO)_2_ (see Fig. 5b). Both effects, decreased charge transfer and orbital energy stabilization, are observed spectroscopically by a shift of transition a to higher energies when going from Cp*Ir(CO)_2_ to CpIr(CO)_2_ along with a substantial increase in oscillator strength. This trend is continued in the more ionic Ir(acac)(CO)_2_ complex. Here, the charge transfer along the 
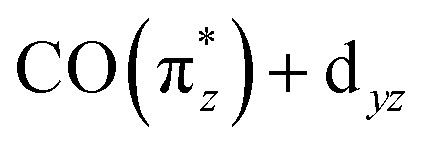
 orbital is further decreased (see Table 2) and its orbital energy further stabilized (see Fig. 5b). This stabilization is so substantial that the 
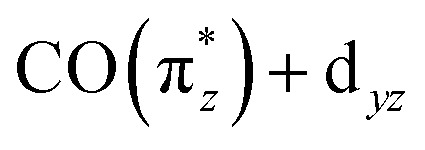
 orbital energy even drops below the d_*z*^2^_ orbital (d_*z*^2^_ now instead being the HOMO in Ir(acac)(CO)_2_). This drop explains the substantially higher transition energy for the 
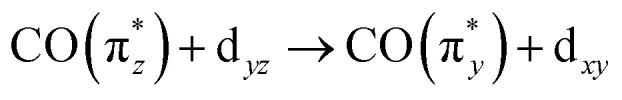
 transition a in Ir(acac)(CO)_2_ compared to Cp*Ir(CO)_2_ and CpIr(CO)_2_. The further reduced charge transfer and correspondingly reduced hybridization of the 
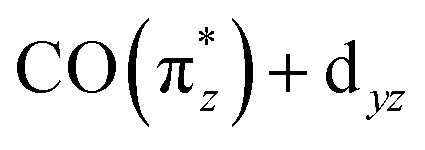
 orbital also explains the stronger intensity of the 
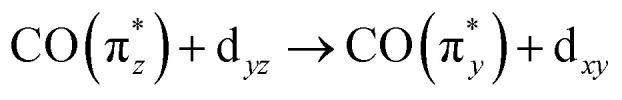
 transition a in Ir(acac)(CO)_2_ compared to the other two complexes.

**Table 2:** Fragment charge decomposition analysis of the three LIr(CO)_2_ complexes (L = Cp*/Cp/acac)

Complex	Electronic charge transfer (in e^−^)		
	L^−^ → Ir^+^(CO)_2_		LIr → (CO)_2_
	Total	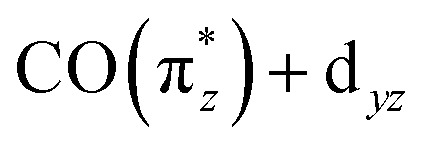	Total
Cp*Ir(CO)_2_	1.064	0.134	0.423
CpIr(CO)_2_	0.948	0.125	0.340
Ir(acac)(CO)_2_	0.715	0.027	0.230

Transitions b, corresponding to excitations from the occupied d_*z*^2^_ orbitals to the unoccupied 
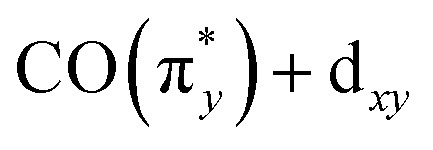
 orbitals in all three complexes, hardly vary with ligand environment either in energy or intensity. This is due to the orientation of this d_*z*^2^_ orbital. In all three complexes, its interaction with the ligands is comparably weak (see the orbital plots in Fig. 5b). It appears as an Ir centered 5d orbital (*i.e.*, large Ir 5d character) unaffected by changing ligands along the Cp*/Cp/acac series, thereby rendering the 
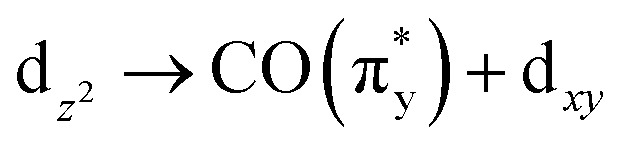
 excitations b substantial and largely non-varying intensities. Transitions c, corresponding to excitations from the occupied d_*x*^2^−*y*^2^_ orbitals to the unoccupied 
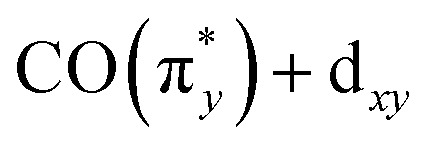
 orbitals in all three complexes, exhibit comparable intensities for Cp*Ir(CO)_2_ and CpIr(CO)_2_, but substantially less for Ir(acac)(CO)_2_. This is due to the shape of the orbital, which points to the center of the Cp*/Cp ring, with which it therefore only weakly hybridizes. For Ir(acac)(CO)_2_, however, the orbital exhibits more hybridization with the oxygen lone pair of the acac ligand leading to substantially reduced oscillator strength of the associated transitions in the range of 5.5 to 7 eV.

On average, the transitions a and b are closest in energy in Ir(acac)(CO)_2_ and, due to the more ionic character of the complex (with higher Ir 5d character of the 
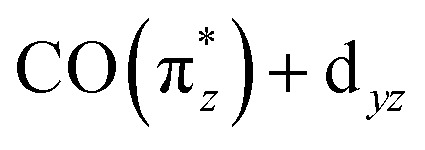
 orbital), have the highest oscillator strengths. This energetic overlap of strong transitions leads to the complex exhibiting the highest intensity in the low-energy-transfer range of the RIXS spectrum between 3.5 and 4.5 eV. In CpIr(CO)_2_ and Cp*Ir(CO)_2_, destabilization of the 
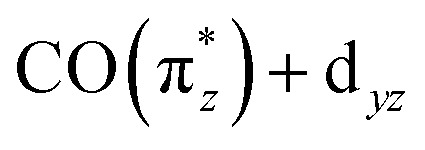
 orbital splits transitions a and b and distributes intensities over a larger energy range. In addition, increased covalency of the 
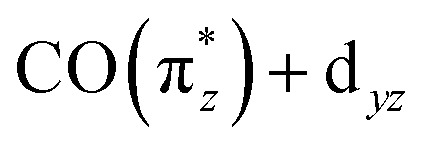
 orbital decreases intensities of transitions a. Together, this causes CpIr(CO)_2_ and Cp*Ir(CO)_2_ exhibiting lower intensities at low energy transfers in RIXS spectrum at 3.5–4.5 eV. The 
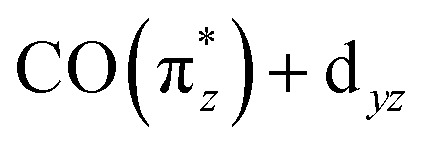
 orbital is most destabilized and most covalent in Cp*Ir(CO)_2_ making this complex having the lowest intensities in this spectral region.

It is important to note that the differences in electronic charge transfer from the L^−^ fragments (L = Cp*/Cp/acac) onto the Ir^+^(CO)_2_ fragment suggested by the charge decomposition analysis directly carry over to differences in charge transfer from the LIr to the (CO)_2_ fragment also shown in Table 2. The highest charge-transfer onto the (CO)_2_ fragment is observed for the Cp*Ir(CO)_2_ complex. The overall charge transfer is then reduced for CpIr(CO)_2_ and even more for Ir(acac)(CO)_2_, which is in full agreement with our interpretation above of the optical, X-ray, and IR absorption data (see discussion of Fig. 2 and 3). The high backdonation capability of complexes with Cp* and Cp groups as ancillary ligands further explains their capability to facilitate C–H activation reactions *via* oxidative addition,^5,6,16,17^ which is not possible with acac-based systems^18^ that are much harder to oxidize due the already charge-depleted metal center.^7^

The differences in the MLCT and d–d manifold of electronic excited states deduced from our optical and RIXS spectra may further provide explanations of the previously observed varying photochemistry of the three complexes.^18,31^ We infer from our findings that optical excitation around 266 nm results in MLCT excitations into the CO π* manifold for Cp*Ir(CO)_2_ and CpIr(CO)_2_. Excitation around 266 nm in Ir(acac)(CO)_2_, instead, results in an acac ligand-centered primary excited state. Ir(acac)(CO)_2_ further exhibits low-lying acac ligand-centered excited states, which are absent in the other two complexes. These states may open parallel non-dissociative excited-state pathways, that compete with dissociative pathways on d–d states. We further infer that, due to the on-average higher energies of d–d states in Ir(acac)(CO)_2_ compared to Cp*Ir(CO)_2_ and CpIr(CO)_2_, fewer d–d excited states are energetically viable decay pathways for the optically generated electronic excited state, possibly further tilting the competition between dissociative and non-dissociative relaxation pathways towards the non-dissociative in Ir(acac)(CO)_2_ compared to Cp*Ir(CO)_2_ and CpIr(CO)_2_. Calculations of the potential energy landscape of valence excited states are in agreement with these conclusions (see SI).

## Conclusion

In summary, we have presented X-ray absorption and VtC RIXS measurements at the Ir L_3_-edge in conjunction with optical absorption spectroscopy of three iridium carbonyl complexes that have previously shown a varying degree of photocatalytic activity towards C–H bond activation. In combination with theoretical modelling, we observe differences in the degree of covalent *vs.* ionic metal–ligand interactions as determined from changes in metal–ligand orbital hybridization and local metal charge densities along the series Cp*Ir(CO)_2_, CpIr(CO)_2_ and Ir(acac)(CO)_2_. This experimental sensitivity to varying metal charge densities thus provides a general tool to assess the electronic factors that determine reactivity towards oxidative addition in C–H activation and other reactions. The here observed differences in metal–ligand interactions are further found to influence the energetics and thus ordering of the valence-excited state manifold, which may impact the efficiency with which optical excitations in the UV regime of the three complexes can trigger CO release as the initial step of photochemical C–H bond activation reactions. Such a comprehensive and orbital-specific access to the excited-state manifold provides an important characterization method to generally understand and tailor the valence electronic structure of metal complexes for a whole range of processes in photocatalysis, as well as phototherapy, that are based on the efficient and photoinduced release of a ligand.

To substantiate our conclusions, we are currently performing time-resolved optical and X-ray studies of these systems complemented by excited-state molecular dynamics simulations. These time-resolved efforts may additionally shed light on the role of CO recombination in the formation efficiency of the reactive metal-monocarbonyl species as well as the role of other ensuing, short-lived reaction intermediates in the overall C–H activation reaction, information which cannot be addressed using the steady-state techniques employed here. Insight inferred from mapping the valence electronic structure with X-ray and optical spectroscopy may then provide a general understanding of the relation between molecular design, metal–ligand covalency and excited-state landscape to better control the efficiency of ligand loss in the design of new catalysts in photochemical C–H activation, phototherapy and photocatalysis.

## Author contributions

R. M. J. and P. W. originated the project concept. R. M. J., M. R., H. Z., N. H, K. J. G., T. K., D. S. and P. W. planned and executed the experiments. R. M. J. analyzed the experimental data. R. M. J and A. B. performed and analyzed the theoretical calculations. R. M. J., A. B. and P. W. wrote the manuscript with input from all the authors.

## Conflicts of interest

There are no conflicts to declare.

## Supplementary Material

SC-OLF-D5SC09924B-s001

## Data Availability

All data supporting the conclusions of this article are included in the main text and the supplementary information (SI). Additional data are available from the authors upon request. Supplementary information is available. See DOI: https://doi.org/10.1039/d5sc09924b.
