# Supplementary material for: Metal–ligand covalency of C–H activating iridium complexes from L-edge valence-to-core resonant inelastic X-ray scattering
Source: Chem Sci. 2026 Feb 16;17(15):7609–19. doi: 10.1039/d5sc09924b (PMC12927629; doi:10.1039/d5sc09924b)
Supplement: SC-017-D5SC09924B-s001 [file SC-017-D5SC09924B-s001.pdf]

Supplementary Information (SI) for Chemical Science.
This journal is © The Royal Society of Chemistry 2026

Supplementary Information:

Metal-Ligand Covalency of C-H Activating Iridium Complexes from L-edge Valence-to-Core Resonant Inelastic X-ray Scattering

Raphael M. Jay,* Ambar Banerjee,* Marco Reinhard, Huan Zhao, Nils Huse, Kelly J. Gaffney, Thomas Kroll, Dimosthenis Sokaras, and Philippe Wernet,*

*Corresponding author. Email: raphael.jay@physics.uu.se, ambar.banerjee@tcgcrest.org, philippe.wernet@physics.uu.se

Input file for geometry optimization of Cp*Ir(CO)$_2$

```
!TPSSh def2-TZVP def2-TZVP/C Opt rijcosx VERYTIGHTSCF
!cpcm(cyclohexane)

%freq
AnFreq true
end

%pal nprocs 16
end

* xyzfile 0 1 Cp_stIrCO2.xyz
```

Input file for generating the orbitals used in the TD-DFT calculation of Cp*Ir(CO)$_2$

```
!B3LYP ZORA ZORA-def2-TZVPP def2-TZVP/C rijcosx TightSCF
!cpcm(cyclohexane)

%basis
NewGTO Ir "SARC-ZORA-TZVP" end
end

%tddft
NRoots 20
end

%pal nprocs 16
end

%maxcore 4000

* xyzfile 0 1 Cp_stIrCO2.xyz
```

Input file for TD-DFT calculation of core and valence excited states of Cp*Ir(CO)$_2$

```
!B3LYP ZORA ZORA-def2-TZVPP def2-TZVP/C rijcosx TightSCF noiter
!cpcm(cyclohexane)

%basis
NewGTO Ir "SARC-ZORA-TZVP" end
end

!moread
%moinp "TDDFT_Cp_stIrCO2.gbw"

%scf
rotate
{2,68,90}
{3,67,90}
{4,66,90}
```

```
end
end

%tddft
tda true
OrbWin[0] = 66,89,90,109
NRoots 1500
end

%pal
nprocs 16
end

%maxcore 4000

* xyzfile 0 1 Cp_stIrCO2.xyz
```

Optimized structures

Cp*Ir(CO)$_2$

Ir	-1.211803	0.182490	-0.314822
C	0.752458	0.437005	-1.468435
C	-1.546401	0.707144	1.438500
C	-0.205088	-0.249882	-2.287888
C	0.971176	-0.379262	-0.306584
C	-0.503273	-1.533319	-1.677480
C	0.223625	-1.614178	-0.458488
C	-2.899717	0.805098	-0.789466
C	-0.673788	0.192741	-3.642378
C	1.958130	-0.095167	0.786819
C	-1.342979	-2.616374	-2.282445
C	0.294286	-2.797304	0.457035
O	-1.737794	1.031782	2.533869
O	-3.944695	1.194435	-1.102711
H	-2.144536	-2.204863	-2.896744
H	-1.794311	-3.245256	-1.513982
H	-0.728782	-3.258526	-2.924183
H	-0.629237	-3.376814	0.429770
H	0.473925	-2.493561	1.488923
H	1.115546	-3.459126	0.158498
H	2.939516	-0.503944	0.522925
H	1.652069	-0.549410	1.730077
H	2.074692	0.977134	0.948593
H	0.006376	-0.180159	-4.415817
H	-0.702483	1.280376	-3.717449
H	-1.670673	-0.189630	-3.864501
C	1.458732	1.713989	-1.803279
H	1.730284	2.264545	-0.901878
H	2.380718	1.507216	-2.359785
H	0.835971	2.362772	-2.420299

$CpIr(CO)_2$

Ir	-1.162138	0.139914	-0.336027
C	0.829342	0.364407	-1.460755
C	-1.503318	0.651318	1.417094
C	-0.126294	-0.269498	-2.305575
C	1.007852	-0.484151	-0.327391
C	-0.465676	-1.550081	-1.739432
C	0.237259	-1.684884	-0.522215
C	-2.840004	0.789585	-0.800929
H	1.303150	1.318001	-1.629311
H	-0.496541	0.118144	-3.242527
H	1.660344	-0.288664	0.510096
H	-1.146331	-2.269383	-2.167397
H	0.193571	-2.525635	0.152164
O	-1.700391	0.967123	2.510848
O	-3.880550	1.189014	-1.104850

$Ir(acac)(CO)_2$

Ir	0.198664	-0.892971	1.232773
C	0.198578	0.375832	2.577402
C	0.168557	-2.219891	2.520160
O	0.198656	1.174302	3.404968
O	0.149884	-3.052089	3.313661
C	0.229051	-0.810263	-2.138262
O	0.229091	0.559936	-0.189710
C	0.235670	0.413620	-1.465785
C	0.213837	-2.065778	-1.526297
O	0.201341	-2.275809	-0.258948
H	0.236838	-0.783605	-3.219236
C	0.210661	-3.312665	-2.364364
C	0.251728	1.701942	-2.238122
H	0.256131	1.530645	-3.313488
H	1.135685	2.280537	-1.957509
H	-0.625083	2.295863	-1.967242
H	0.220710	-3.084397	-3.429063
H	-0.676319	-3.905815	-2.127187
H	1.084713	-3.919219	-2.113495

Whiteline intensity analysis

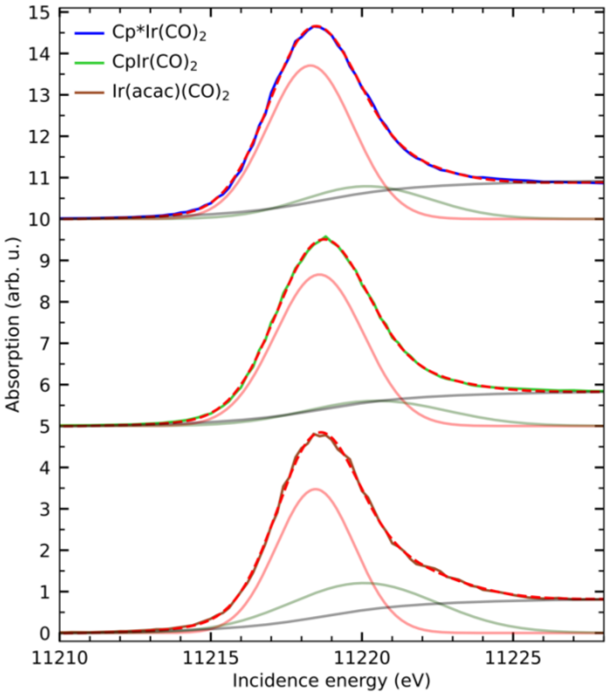

Fig. S1 Closeup of the whitelines of the three complexes. Each whiteline is fitted with the sum of two Gaussian functions as well as an arctangent function, which is centered at the maximum of the whiteline and broadened with the 5.24 eV lifetime broadening of the Ir 2p core-hole. The integral of the sum of the two Gaussians is then used as an estimate of the total whiteline intensity for each of the three complexes.

Whiteline shift

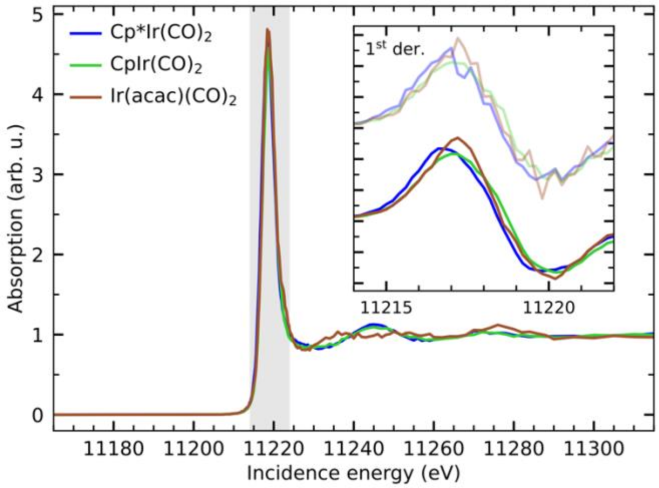

Fig. S2 HERFD X-ray absorption spectra of $Cp^*Ir(CO)_2$, $CpIr(CO)_2$ and $Ir(acac)(CO)_2$ measured at the Ir L_3-edge. The inset shows the unsmoothed 1st derivative of the absorption spectra in the area of the whiteline (top) as well as the smoothed 1st derivative of the spectra (bottom) as shown in the main text for better visualization of the relative shifts. The magnitude of the shifts has been determined from both the unsmoothed as well as smoothed derivatives yielding the same values.

Potential energy surfaces

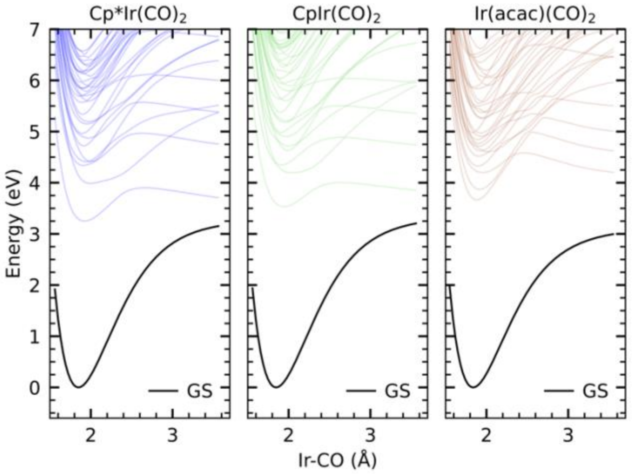

Fig. S3 Calculated potential energy surfaces of valence excited states as a function of the Ir-CO bond lengths for the three complexes.

Additional experimental details

HERFD spectra have been recorded with a total exposure time of 0.5s, 3.5s and 10s per point for Cp*Ir$(CO)_2$, Cp*Ir$(CO)_2$, and Ir(acac)$(CO)_2$, respectively. The incidence energy was moved as follows:

11165 eV - 11195 eV (5 eV step size)
11195 eV - 11205 eV (1 eV step size)
11205 eV - 11210 eV (0.25 eV step size)
11210 eV - 11224 eV (0.2 eV step size)
11224 eV - 11230 eV (0.5 eV step size)
11230 eV - 11260 eV (1 eV step size)
11260 eV - 11290 eV (2 eV step size)
11290 eV - 11500 eV (5 eV step size)

The RIXS maps were measured with a total exposure of 0.5s, 3.5s and 5s time per point in each RIXS map for Cp*Ir$(CO)_2$, Cp*Ir$(CO)_2$, and Ir(acac)$(CO)_2$, respectively. The incidence energy was moved in the range of 11210 eV to 11225 eV in steps of 0.25 eV. At each incidence energy, the emission energy was scanned in the range of 11204 eV to 11218 eV in steps of 0.25 eV.

Identification of 5d orbitals

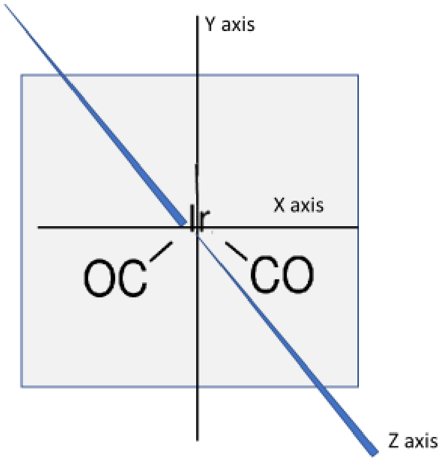

Fig. S4 Coordinate system based on the $Ir(CO)_2$ structural motif used for identifying the 5d orbitals.

The $Ir(CO)_2$ structural motif is common to all the three complexes studied here and was therefore used to define a common coordinate system as shown in Fig. S4. The C-Ir-C atoms in the $Ir(CO)_2$ moiety form a plane which is unique to all three complexes and we considered this plane to be the xy plane. The y axis is taken to be along the direction of the perpendicular bisector of the C-Ir-C angle in the $Ir(CO)_2$ moiety. As a result, the LUMO can be designated as a d_{xy}-derived orbital as shown in the main text. This also gives the description of the z axis, i.e., in a direction perpendicular to the xy plane. Based on this, the d_{z^2}-derived orbital (shown in the main text) can be defined along with the $d_{x^2-y^2}$ (shown below), as well as d_{xz} (shown below) and d_{yz}-derived orbitals (shown in the main text).

**Complex**	$\mathbf{d_{xz}}$	$\mathbf{d_{x^2-y^2}}$
$Cp^*Ir(CO)_2$	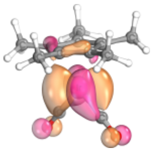	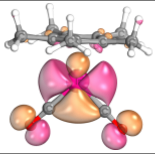
$CpIr(CO)_2$	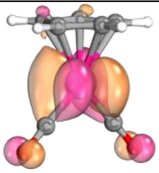	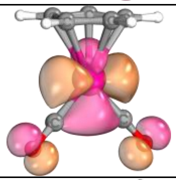
$Ir(acac)(CO)_2$	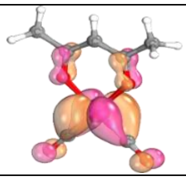	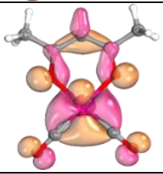

Orbital assignment of optical transitions

Complex	Wavelength (nm)	Dominant Kohn-Sham orbitals
$Cp^*Ir(CO)_2$	271	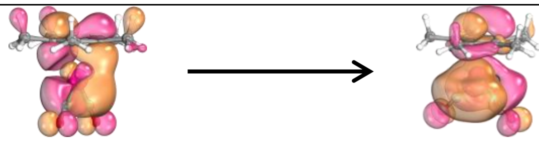
$CpIr(CO)_2$	253	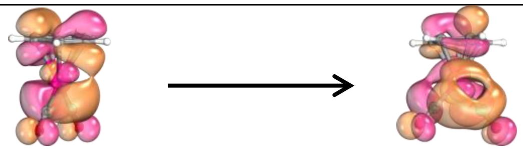
$Ir(acac)(CO)_2$	309	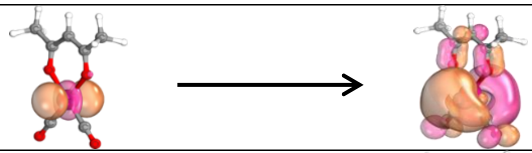
	245	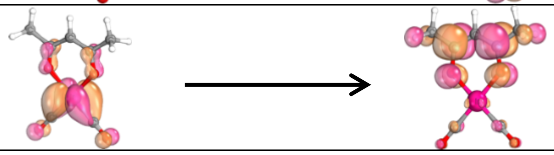

Natural Transition Orbital analysis of RIXS transitions

Complex	Energy (eV)	Transition character	
$Cp^*Ir(CO)_2$	3.31	>0.99	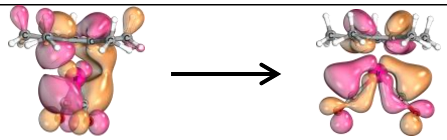
	4.38	>0.99	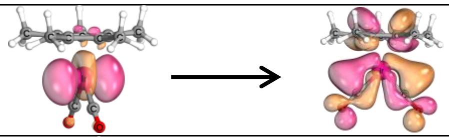
	5.25	0.74	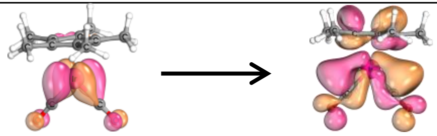
		0.22	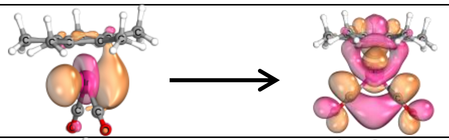
	5.77	0.56	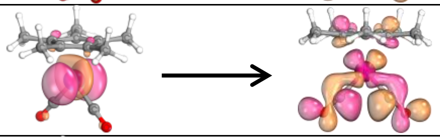
		0.19	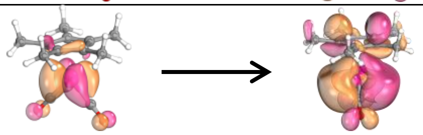

		0.14	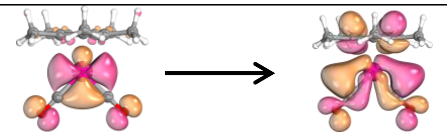
	6.24	0.77	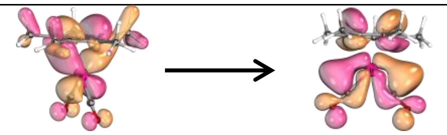
		0.12	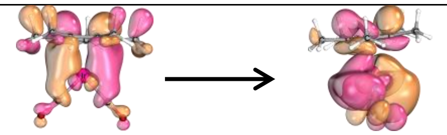
	6.52	0.41	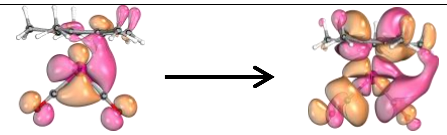
		0.34	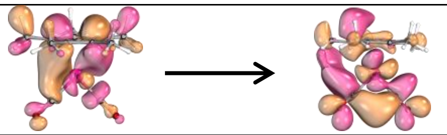
CpIr(CO)$_2$	3.60	>0.99	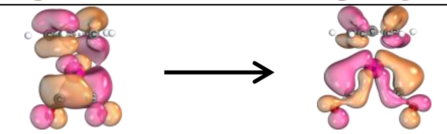
	4.39	>0.99	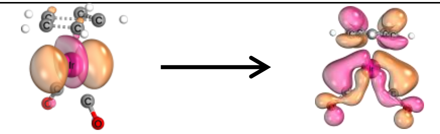
	5.34	0.64	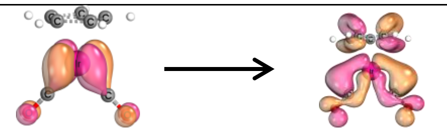
		0.33	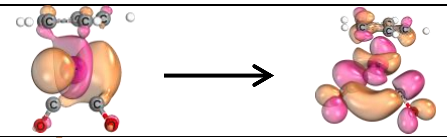
	5.77	0.83	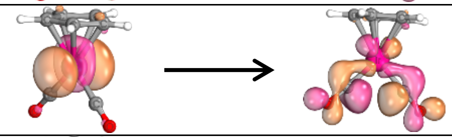
	5.96	0.62	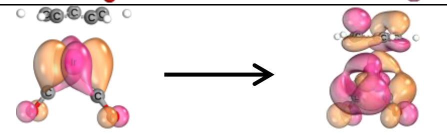
		0.21	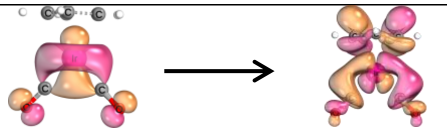
		0.11	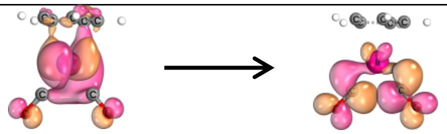

	6.48	0.51	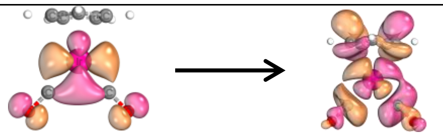
		0.23	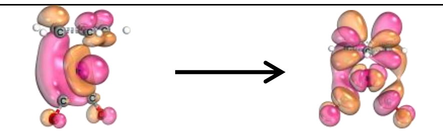
		0.19	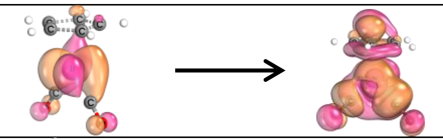
Ir(acac)(CO)$_2$	4.28	>0.99	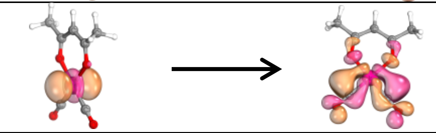
	4.75	>0.99	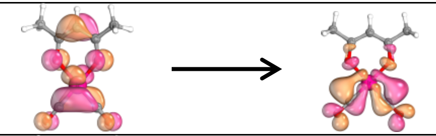
	4.99	0.68	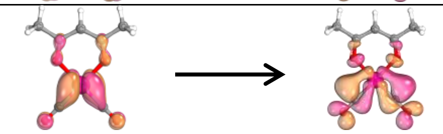
		0.24	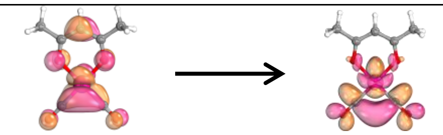
	5.63	0.60	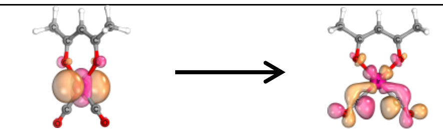
		0.23	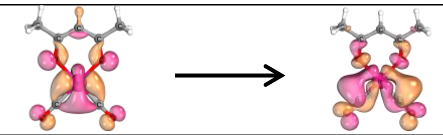
	5.76	0.43	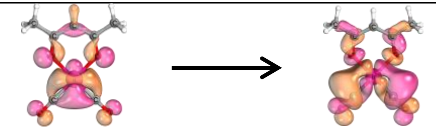
		0.28	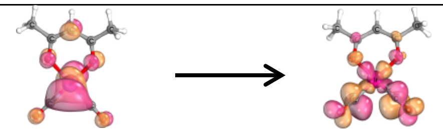
		0.20	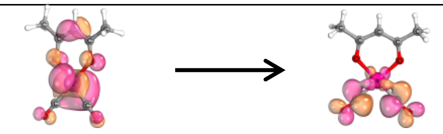
	6.49	0.79	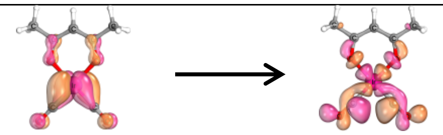

		0.15	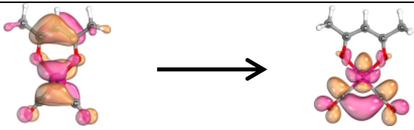
	6.50	0.48	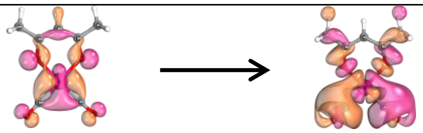
		0.42	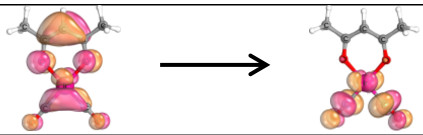
